# Magnetic Hyperthermia Nanoarchitectonics via Iron Oxide Nanoparticles Stabilised by Oleic Acid: Anti-Tumour Efficiency and Safety Evaluation in Animals with Transplanted Carcinoma

**DOI:** 10.3390/ijms23084234

**Published:** 2022-04-11

**Authors:** Oleg A. Kulikov, Mikhail N. Zharkov, Valentin P. Ageev, Denis E. Yakobson, Vasilisa I. Shlyapkina, Andrey V. Zaborovskiy, Vera I. Inchina, Larisa A. Balykova, Alexander M. Tishin, Gleb B. Sukhorukov, Nikolay A. Pyataev

**Affiliations:** 1 Institute of Medicine, National Research Ogarev Mordovia State University, Bolshevistskaya Str. 68, 430005 Saransk, Russia; oleg-kulikov-84@mail.ru (O.A.K.); mikhail.zharkov.92@mail.ru (M.N.Z.); valeageev@yandex.ru (V.P.A.); ykbsn@mail.ru (D.E.Y.); shlyapkina.98@mail.ru (V.I.S.); v.inchina@yandex.ru (V.I.I.); larisabalykova@yandex.ru (L.A.B.); 2 Department of Pharmacology, A.I. Yevdokimov Moscow State University of Medicine and Dentistry, Delegatskaya Str. 20, 127473 Moscow, Russia; azabor@mail.ru; 3 Faculty of Physics, M.V. Lomonosov Moscow State University, Leninskie Gory 1-2, 119991 Moscow, Russia; tishin@amtc.org; 4 Pharmag LLC (AMT&C Group), Promyshlennaya Str. 4, 142190 Troitsk, Russia; 5 School of Engineering and Materials Science, Queen Mary University of London, Mile End Road, London E1 4NS, UK; g.sukhorukov@qmul.ac.uk; 6 Skolkovo Institute of Science and Technology, Bolshoy Boulevard 30, Bld. 1, 143025 Moscow, Russia

**Keywords:** iron oxide nanoparticles, oleic acid, toxicity, magnetic hyperthermia, W256 carcinoma, anti-tumour activity

## Abstract

In this study, we developed iron oxide nanoparticles stabilised with oleic acid/sodium oleate that could exert therapeutic effects for curing tumours via magnetic hyperthermia. A suspension of iron oxide nanoparticles was produced and characterised. The toxicity of the synthesised composition was examined in vivo and found to be negligible. Histological examination showed a low local irritant effect and no effect on the morphology of the internal organs. The efficiency of magnetic hyperthermia for the treatment of transplanted Walker 256 carcinoma was evaluated. The tumour was infiltrated with the synthesised particles and then treated with an alternating magnetic field. The survival rate was 85% in the studied therapy group of seven animals, while in the control group (without treatment), all animals died. The physicochemical and pharmaceutical properties of the synthesised fluid and the therapeutic results, as seen in the in vivo experiments, provide insights into therapeutic hyperthermia using injected magnetite nanoparticles.

## 1. Introduction

In the past two decades, cancer research has led to significant advances in the treatment of the disease, including synthesis of new anticancer drugs, development of new methods of physical impact on tumours and their combination with chemotherapy. Hyperthermia (HT) is a promising approach for cases in which physical methods are strongly envisaged to help [1]. HT is based on the effect of alternating electromagnetic fields of different frequencies [2,3,4,5] on tumour tissue, which leads to local heating and death of tumour cells. Tumour cells are considered to be more thermolabile than normal cells; this has been confirmed by several studies [6,7,8]. Contrastingly, some reports state that the difference in temperature sensitivity of normal and tumour tissues is debatable, at least for some tumour types [9,10,11,12].

In addition, a majority of human tumours are characterised by the absence of spatial ordering of tumour cells and the stromal component, which makes it impossible to heat them uniformly over a particular location [13,14]. Hence, excessive heating is required, which limits the potential of physically induced remote heating, such as that for methods based on microwave radiation [15]. In such heat treatments, the surrounding healthy tissues are warmed up as well, resulting in their necrosis [16,17]. A local effect on heating of the tumour can be realised via magnetic hyperthermia (MHT) by a targeted administration of magnetic nanoparticles into the tumour tissue and heating with an alternating magnetic field [18,19]. In this regard, the development of magnetic particles that can be used for MHT in vivo is a topic of particular interest for nanoparticle delivery. The key parameter that determines the applicability of magnetite particles for MHT is the specific absorption rate (SAR) of the material, which defines the properties to convert alternating magnetic irradiation into heat release [20].

In addition to SAR, there are other important factors to be considered for the applicability of MHT. The necessary conditions for the suitability of a magnetic fluid for biomedical applications are biocompatibility and low toxicity. Recently, several compositions with high SAR values have been proposed. However, only a few reports show the effectiveness of the developed compounds in vivo. This is because, in order to increase SAR, magnetic nanoparticles should be incorporated with additional ions, such as zinc, manganese, cobalt, nickel, rare earth elements and other heavy metals [21,22,23,24,25].

Some reports have described compounds with high SAR values, but these are quite toxic and cannot be used in animals and humans [26,27]. In addition, to increase the efficiency of MHT, the encapsulation of magnetically active particles in various structures, such as microcapsules, has been proposed. However, this increases their absorption by cells, leading to increased cytotoxicity [28,29].

There are very few publications that report the fabrication of a commercial, clinically applicable magnetic fluid for MHT [30,31].

Currently, iron oxide nanoparticles are most promising with respect to pharmaceutical applications, owing to their low toxicity [27,32,33]. Because the SAR of iron oxide is not very high, an alternative compound with a sufficiently high concentration of iron (approx. 2–3 M) as well as with sufficient stability for storage purposes needs to be developed. NanoTherm^®^ magnetic fluid developed by MagForce is such a compound, which is based on a suspension of iron oxide nanoparticles. In these fluids, the problem of stability of the highly concentrated suspension is solved using silicon compounds, particularly aminosilane [32]. This compound provides a stable suspension with an iron concentration of up to 5 M. However, disadvantages mainly related to low biodegradability still exist. For successful treatment, an additional operation is required to remove the injected particles [34].

In this study, we explored the use of oleic acid and its sodium salt as particle stabilisers. These compounds are often used for the synthesis of ferromagnetic colloids because their carboxyl groups can bind firmly to the surface of nanoparticles and form a double molecular layer on them in aqueous solutions [35,36,37]. This covering layer prevents the oxidation of iron and the agglomeration of particles. In addition, oleic acid is biocompatible and completely metabolised. Nanoparticles of iron oxide coated with oleic acid were prepared [38,39], and their cytotoxicity was studied in various cell cultures. The aim of our work was to study the effect of MHT on an in vivo tumour model. We synthesised a stable, highly concentrated magnetic fluid using iron oxide nanoparticles stabilised with oleic acid and carried out an in vivo study on rats with induced tumours. First, we examined the acute toxicity of the synthesised fluid in vivo and its effect on haematological and biochemical blood parameters. Second, we investigated the local irritant effect of this fluid to assess the possibility of intra-tumoural administration. Then, we evaluated the anti-tumour effect of MHT using a fabricated suspension in rats with transplanted Walker 256 (W256) carcinoma. The inhibitory effect of the suspension on tumour growth and the survival rate of the animals were evaluated.

## 2. Results and Discussion

### 2.1. Characteristics of Magnetic Nanoparticles

**Figure 1:**
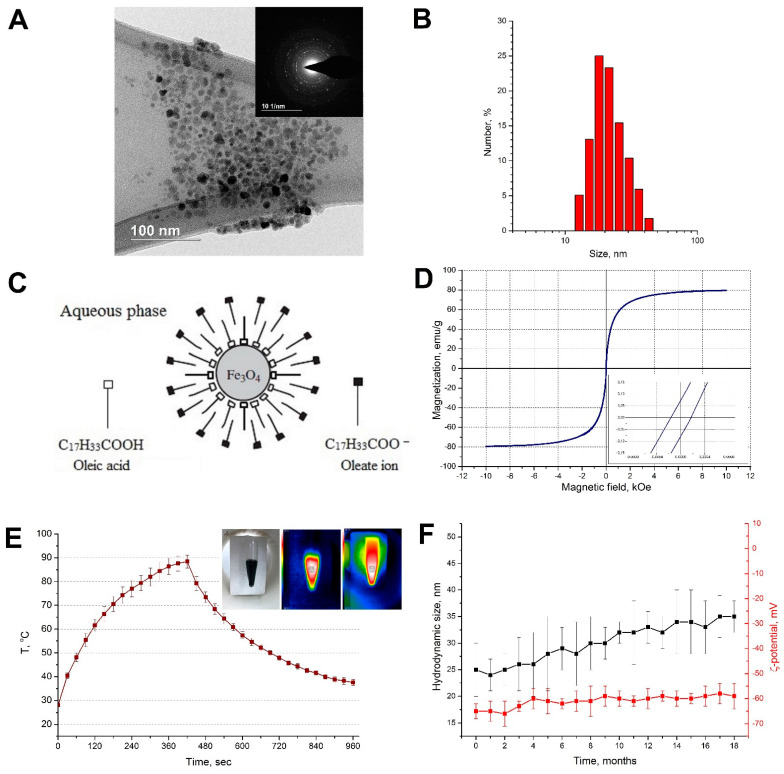
(**A**) Transmission electron micrograph of Fe_3_O_4_ nanoparticles; (**B**) size distribution of Fe_3_O_4_ nanoparticles; (**C**) scheme of the structure of a nanoparticle; (**D**) magnetisation curves of Fe_3_O_4_ nanoparticles; (**E**) time–temperature curve of Fe_3_O_4_ nanoparticles in the aqueous phase (112 mg Fe/mL) under AMF (100 kHz, 8 kA/m), inset-IR images of the test tube with Fe_3_O_4_-OA-NP inside the coil; (**F**) hydrodynamic size and ζ-potential of Fe_3_O_4_-OA-NP during 18 months of storage.

We obtained an aqueous, highly concentrated suspension of Fe_3_O_4_ nanoparticles stabilised by neutral molecules of oleic acid and oleate ions. The modification of the synthesis method using ethanol increased the solubility of oleic acid and sodium oleate, and therefore better stabilised the magnetic nanoparticles. In addition, ethanol has a lower boiling point (~78 °C) than water, which allows rapid and accurate evaporation of the medium to increase the concentration of particles in the suspension and maintain their stability. Figure 1 shows the properties of the synthesised particles.

The mass concentration of iron in the suspension was 112 mg Fe/mL. Figure 1A shows that the particles were sufficiently monodispersed and had a close to spherical shape. The nanoparticles had an average size of 8.7 ± 3.1 nm. According to the results of the DLS study (Figure 1B), the average particle size was 25 ± 8 nm. The larger hydrodynamic size of the particles (in comparison with the TEM data) was due to the formation of small agglomerates in the aqueous solution. Typically, such particles have an iron oxide core and a two-layer shell. The inner layer comprises oleic acid bound to the core, and the outer layer is formed by oleate ions oriented toward the aqueous phase by the hydrophilic part (Figure 1C). The ζ-potential of the magnetite nanoparticles was –65 ± 3 mV at pH 7.8 (before the measurements, the magnetic suspension was diluted 10 times with deionised water).

Figure 1D shows the magnetisation curve for the Fe_3_O_4_-OA-NP suspension. The small hysteresis loop indicates the superparamagnetic properties of the suspension. In this case, the value of residual magnetisation was 0.073 emu/g, and the low-value coercive force was 0.21 Oe (inset in Figure 1D). The saturation magnetisation for the Fe_3_O_4_-OA-NP suspension was 79.5 emu/g.

The synthesised magnetic suspension had a high thermal energy transfer capability. When exposed to an AMF (8 kA/m, 100 kHz), the temperature was elevated by approximately 60 °C in 7 min (Figure 1E).

The hydrodynamic size and ζ-potential for Fe_3_O_4_-OA-NP during 18 months of storage showed that the nanoparticles remained stable throughout the observation period; the average hydrodynamic diameter of the particles increased by approximately 10 nm (Figure 1F). In addition, no stratification of the magnetic suspension was observed. The SAR of the magnetic suspension decreased to 9 W/g Fe after the storage period. This decrease in SAR was most likely associated with a change in the magnetic properties of the nanoparticles. Fe^2+^ ions in the Fe_3_O_4_ structure can oxidise and change the surface anisotropy of the particles [40].

The resulting magnetic fluid with iron oxide nanoparticles had characteristics that allowed it to be used for medical purposes. Therefore, we conducted toxicological studies on the fluid.

### 2.2. Acute Toxicity

The toxicity parameters of the synthesised magnetic fluid when administered intraperitoneally are listed in Table 1.

The dose dependence of the induced lethality is shown in Table 1 (in absolute numbers) in the supplementary material (Figure S1, Table S1) (with calculated probit curves).

For the intramuscular injection of the Fe_3_O_4_-OA-NP magnetic fluid, we observed no deaths throughout the observation period. In most animals, resorption of the infiltrate at the site of the injected nanoparticles was observed 30 days after intramuscular injection. The hind limb function was preserved in all mice. All mice in this study received an average single dose of 5600 mg Fe/kg of Fe_3_O_4_-OA-NP. Therefore, the Fe_3_O_4_-OA-NP magnetic fluid could be classified as a low-toxicity compound when administered intraperitoneally and a practically non-toxic or relatively harmless compound when administered intramuscularly [41].

Histological examination after intraperitoneal and intramuscular injection of Fe_3_O_4_-OA-NP showed no signs of disturbance in the morphology of the internal organs of mice during macro- and microscopic assessment (described in the Supplementary Materials, Figures S2 and S3).

### 2.3. Local Irritant Effect and Effect on Blood

#### 2.3.1. Local Irritant Effect

On the seventh day after intramuscular injection, Fe_3_O_4_-OA-NP persisted at the injection site, and there was some increase in the right thigh volume due to oedema. However, there were no marked signs of inflammation or necrosis.

**Figure 2:**
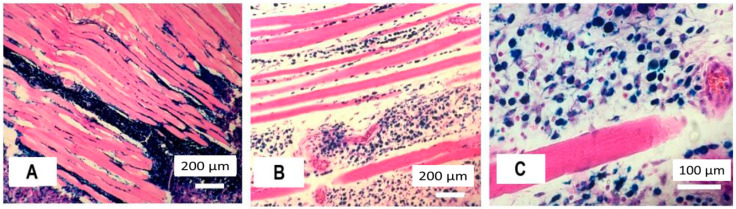
Histological changes in the rat hip muscle on the seventh day after injection of Fe_3_O_4_-OA-NP with a magnetite dosage of 233 mg Fe/kg. Site around the central injection (**A**); 10 mm from region of injections (**B**,**C**); impregnation of perimysium (**C**) with ferrofluid.

Microscopic examination of the intact femoral muscles (left paw) showed the correct arrangement of muscle fibres with a characteristic transverse striation. No signs of pathological processes were observed. The Perls’ reaction was negative. In the muscles of the right thigh, iron-oxide-containing structures resembling dark blue conglomerates were observed (Figure 2A).

A detailed examination showed that Fe_3_O_4_-OA-NP was localised intracellularly. Cells containing Fe_3_O_4_-OA-NP were present among tissue phagocytes. Among the macrophages, the contours of fibroblasts were visualised; they formed loose reticular structures (Figure 2C). Fe_3_O_4_-OA-NP completely filled the cytoplasm of phagocytes, and Prussian blue gave it a deep blue colour; the nucleus was not visible. Loosening of the muscle fibres was noted (Figure 2B). Some of the fibres were sinuous (Figure 2A). The muscle fibres were spaced apart due to oedema of the loose connective tissue of the endomysium. At a magnification of 400× (Figure 2C), the transverse striation of muscle fibres was visualised, indicating the preservation of the contractile function. There were no foci of sarcoplasmic lysis or fibre necrosis.

At a distance from the injection site (in the adjacent fascial case), the concentration of iron oxide particles was lower. Accordingly, the phagocyte cytoplasm was free of iron.

The perimysium space was abundantly infiltrated by leucocytes (Figure 2B). Some of the cells contained Fe_3_O_4_-OA-NP in the cytoplasm. In places where magnetite emerged under the fascial loose connective tissue (epimysium), intense impregnation of various morphological structures was observed. In particular, the adipocyte walls of the epimysium fatty layers showed intense blue coloration. The outer layer of the adventitia of the arteries was also well saturated with magnetic fluid. The high sorption of Fe_3_O_4_-OA-NP by the epimysium and perimysium may have been due to the stabilisation of magnetic nanoparticles with oleic acid.

#### 2.3.2. Effect on Blood

The changes in haematological parameters and biochemical blood tests on the seventh day after the intramuscular injection of Fe_3_O_4_-OA-NP are presented in the Supplementary material (Tables S2 and S3). Statistically significant changes were found in such parameters as WBC, ALP, AST and serum iron. However, both at the initial stage and after the introduction of the magnetic fluid, these indicators did not go beyond the normal range. For a real assessment of the clinical and physiological significance of these effects, an increase in the number of observations or an investigation in special condition is required.

It should be noted that the toxicity of the synthesised fluid could be determined both using the shell (oleic acid/sodium oleate) and the iron oxide core. Moreover, it was highly dependent on the route of administration. Free oleic acid is known to be toxic after intravenous administration, causing acute lung injuries, such as adult respiratory distress syndrome. The mechanism of this damage is based on the destruction of the endothelial barrier of the pulmonary capillaries, which leads to severe pulmonary oedema [42].

It is also known that ionic iron is more toxic than its crystalline form because of its higher intracellular anti-enzymatic activity, and ferrous ions (Fe^2+^) are 3–4 times more toxic than ferric ions (Fe^3+^). The mechanism of iron toxicity is associated with the generation of reactive oxygen species (ROS) and the production of malonic dialdehyde, which inactivates many types of enzymes and damages the cell membrane [43]. In addition, iron can disrupt the activity of enzymes by binding with their SH and NH groups [44].

In this study, the toxic effects were not significant. The low toxicity can be explained by the peculiarities of the introduction route. In the case of intraperitoneal and intramuscular administration, particles are not injected into the systemic circulation but remain at the injection site and are then gradually resorbed. The resorption occurs due to the absorption of particles by macrophages and subsequent digestion in lysosomes. Oleic acid is partially transported to the liver, where it is metabolised by beta-oxidation, whereas iron oxide nanoparticles are converted into an ionic form and excreted by the kidneys [45]. The rate of resorption does not seem to overload the enzyme and detoxification systems, as no increase in iron concentration in the blood or toxic lung damage associated with oleic acid were observed. It is notable that in the case of intraperitoneal administration, the toxicity was slightly higher than that in the case of intramuscular administration. Since iron oxide nanoparticles cannot be transported directly through the cell membrane [46,47], the rate of their resorption from the peritoneal cavity is unlikely to be higher than that from muscle tissue. However, with this route of administration, the toxic effects can increase due to the interaction of particles with the organs of the abdominal cavity, primarily the intestines and liver.

It is also remarkable that we did not observe a significant local irritant effect. This was probably due to the neutralisation of the acid during the synthesis of particles in the alkaline medium and the shielding of the aggressive iron oxide core by the lipid shell [46].

### 2.4. Antineoplastic Effect of MHT

#### 2.4.1. Study of Heating and Growth of W256 Adenocarcinoma

**Figure 3:**
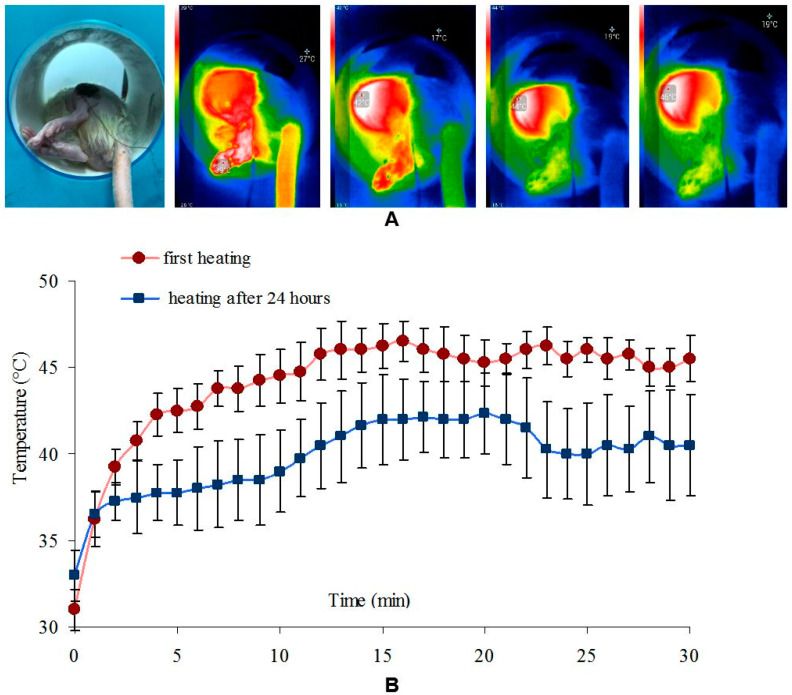
(**A**) IR visualisation of the heating process of the tumour injected with Fe_3_O_4_-OA-NP; (**B**) dynamics of heating of the tumour injected with Fe_3_O_4_-OA-NP during the first and second (after 24 h) exposure to a magnetic field.

When the tumour was heated immediately after the injection of magnetic fluid containing Fe_3_O_4_-OA-NP, the target temperature (45 °C) was reached after 12 min (Figure 3). Heating the tissues from 30 to 40 °C took only 3 min. During the second HT session, after 24 h, the maximum temperature was 42.3 ± 1.3 °C. It was also possible to reach 40 °C in 12 min (Figure 3B).

As shown in Figure 3B, repeated exposure to the magnetic field after 24 h did not cause sufficient heating of the injection site for effective HT.

#### 2.4.2. Tumour Growth

**Figure 4:**
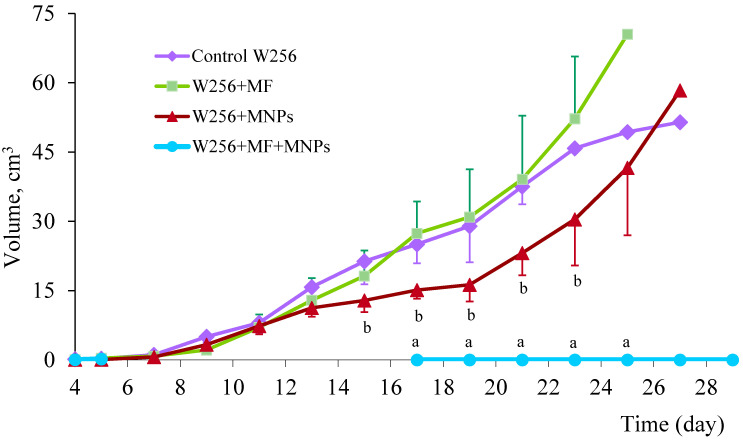
Dynamics of tumour node growth of Walker 256 carcinoma. The tumour was not detected in the MHT group (a—tumour node volume in the MHT group is statistically significantly different from other ones, *p* < 0.05). There was a temporary inhibition of tumour growth in the ferrofluid control group (b—statistically significantly different between MNPs group and control one, *p* < 0.05).

The tumour growth curves are shown in Figure 4. The greatest interest was aroused by the dynamics of tumour growth in the MHT group.

In this group, oedema developed at the site of injection, and it was not possible to measure the node until the 15th day of the experiment. At this time point, tumour growth was completely absent in five animals, and the animals already showed no femoral oedema. All the animals had ulceration with necrotic masses at the site of the tumour node. In two animals of the MHT group, tumour growth resumed. In one of them, a re-growth of a tumour node at the implantation site was observed, but after a single re-injection of Fe_3_O_4_-OA-NP and MHT, the growth did not resume. In the second animal with relapse, the tumour continued to grow outside the area of injection, and this animal died on the 21st day. Healing of the ulcerative-necrotic defect in the MHT group occurred for up to 30 days. A dense connective tissue scar remained at the healing site.

In the other groups, node growth was observed throughout the observation period. The tumour volume in the groups not subjected to HT for the first 7 days increased 3–4 times every 2 days. From the 11th day, the growth of the node slowed down but continued until the death of the animals.

#### 2.4.3. Blood Analysis

The values of the haematological and biochemical blood parameters in the control and MHT groups are presented in the supplementary material (Tables S4 and S5). On the 19th day, we observed a decrease in the level of red blood cells and haemoglobin, as well as an increase in leucocyte levels in both the control and experimental groups. However, in the MHT group, anaemia and leucocytosis were not as significant as in the control group. A more pronounced presence of leucocytosis and anaemia in the control group can be explained by the development of inflammation in the tumour node and tumour-associated intoxication.

Among the biochemical parameters of blood, attention should be paid to high creatinine, urea and bilirubin values, which were especially pronounced in the MHT group. On the 40th day of the experiment, in the MHT group, the levels of urea and creatinine decreased by three and two times, respectively. The increase in creatinine and urea levels can be explained by the fact that, during HT, the tumour and areas of muscle tissue surrounding it were damaged by heating, which led to the appearance of protein breakdown products in the blood. The reason for the increase in bilirubin levels remains unclear because it cannot be explained by liver damage, as the values of ALT and AST (liver damage markers) in the MHT group were not different from those in the normal group.

#### 2.4.4. Histological Observation of Region of Treatment

**Figure 5:**
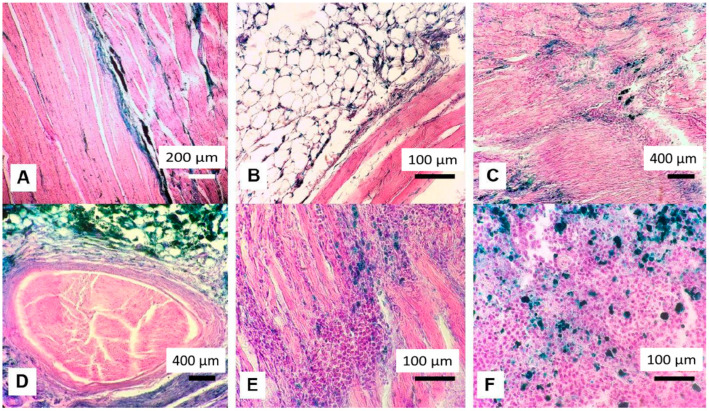
Pathological observation of region of treatment in magnetic hyperthermia (MHT) group (**A**–**E**) and ferrofluid control group (**F**). On the 40th day of the experiment, the Perls’ reaction was positive in the muscle and adipose tissue of rats from the MHT group. Prussian blue was located in the endomysium of muscle tissue and intercellular septa of adipocytes (**A**,**B**). At the sites of tumour neutralisation, areas of dense fibrous connective tissue are found (**C**), and these may have a capsule (**D**). On the 40th day, one individual from the MHT group had tumour cells at the site of tumour inoculation, and these were placed intramuscularly (**E**). On the 29th day, magnetite was detected in tumours of rats from the ferrofluid control group, within cancer cells and macrophages. These cells had a blue cytoplasm (**F**).

Perls’ staining of the regions of paws exposed to HT showed the presence of iron compounds (Figure 5A–D). Tumour cells in the region of treatment were not found in five of the six surviving animals. In one rat from the MHT group, cancer cells were discovered at the site of tumour inoculation. They were located among the muscle fibres (Figure 5E). These cancer cells showed a ‘silent’ recurrence of W256 adenocarcinoma in this animal, which may cause the development of a new tumour after some time.

As in the case of the study of the local irritant effect, the magnetic fluid was located diffusely in the muscle tissue, penetrating the endomysium and evenly staining it blue (Figure 5A).

In muscle tissue and subcutaneous fat, macrophages with cytoplasm that reflected the Perls’ colour were found (Figure 5A,B). Foci with a higher intensity of Prussian blue colour were observed; these can presumably be the places where the needle was inserted when Fe_3_O_4_-OA-NP was injected. Muscle fibres in contact with the magnetic fluid did not show signs of membrane damage and retained their transverse striations. The muscles had local foci of fibrous connective tissue (microscars). The areas of scarring were located near the muscle fibres (Figure 5C). Some foci of connective tissue had a capsule (Figure 5D). These foci may have formed at the sites of the dead tumour tissue. The adipocytes surrounding the muscle had a cell membrane accumulating the Perls’ stain (Figure 5B).

In animals of the ferrofluid control group, magnetite was located intracellularly in the tumour tissue (Figure 5F). The cells containing magnetite were macrophages and adenocarcinomas. The nanoparticles inside tumour cells and macrophages were visualised by reaction of the formation of Prussian blue. Tumour cells with the corresponding colour accumulated magnetite nanoparticles. Against the background of the cell contours, Prussian blue granules are visualised; there are no granules in the intercellular space. This allows us to draw a conclusion about the intracellular localisation of objects containing Fe^2+^ (in this case, conglomerates of nanoparticles).

The intracellular localisation of magnetite can enable a more pronounced cytopathic effect of HT, as magnetite may heat up in the cytoplasm, damaging the enzyme systems [48,49]. Magnetite coming into contact with the cell membrane from the outside causes less damage, and the cell can survive under such conditions. Considering the fact that cancer cells have an active chromatin devoid of a nuclear membrane [50], tumour cells may become more sensitive to HT than healthy cells upon intracellular localisation of magnetite [51].

#### 2.4.5. Influence of HT on Survival Period of Rats

**Figure 6:**
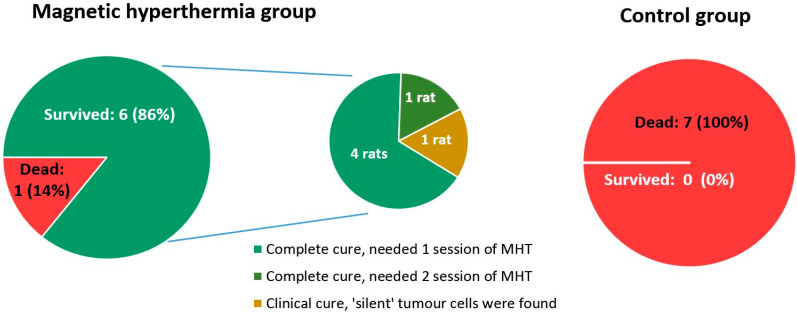
Survival rate of rats with Walker 256 carcinoma treated with and without magnetic hyperthermia. For control groups, only one graph is shown, because mortality was similar for all. Differences in survival rates between the MHT group and other groups are statistically significant (*p* < 0.05).

The average life expectancy in the control group without any influences was 20.9 ± 1.5 days; for the W256 + MF and W256 + MNPs groups, it was 23.7 ± 1.5 and 24.0 ± 1.9 days, respectively. The death of animals was observed on the 19th day after tumour transplantation. From the 23rd day, survival in the MHT group was significantly greater than that in the control group, and from the 25th to 29th day, survival was significantly greater than that in the W256 + MF and W256 + MNPs groups (Figure 6). In the MHT group, only one animal died within 40 days.

In total, six out of the seven animals in the experimental group survived, giving an 85.7% success rate, whereas five out of seven rats were completely cured (71.4%). Among the surviving animals, one had a recurrence in the primary tumour node. This was probably associated with insufficient heating of a certain area of the tumour. Additional injection of magnetic fluid and a session of MHT enabled complete tumour degradation and survival of the animal. Cases of unsuccessful therapy are of particular interest. In the experimental group, one animal died because of the spread of the tumour along the metastatic pathways. In this case, a secondary tumour can be considered a regional metastasis, for which the subsequent use of MHT becomes anatomically and technically impossible. Differences in survival rates between the MHT group and other ones were statistically significant (*p* < 0.05).

Another interesting finding was the discovery of so-called ‘silent’ tumour cells without visible tumour growth in one animal. On the one hand, this indicates a fairly high resistance of W256 to HT, possibly due to its low differentiation [50]. On the other hand, the presence of silent cancer cells may be a consequence of the effect of HT on the cell cycle of this tumour. HT may inhibit the G2–M [52] transition and slow down proliferation or lead to the arrest of the cell cycle in the G1 phase [53]. In this case, cancer acquires a latent course with visible clinical recovery, but relapses occur sooner or later.

One way or another, the experiments demonstrated the high proliferative activity of W256 and the possibility of survival of its cells even under the condition of intense uniform impregnation with magnetic nanoparticles followed by heating to the coagulation temperature. Hence, we believe that in the case of aggressive tumours, namely poorly differentiated, small-cell, intensively proliferating epithelial cancers with high early metastatic activity, hard heating with complete coagulation of the tumour node should be the strategy of choice. The selective destruction of a tumour using this approach can be achieved by the targeted introduction of magnetic particles using modern navigation technologies [54]. The use of gentle low-temperature techniques can lead to an incomplete reduction in the tumour and its recurrence.

## 3. Materials and Methods

### 3.1. Reagents

Iron(III) chloride, iron(II) chloride, oleic acid and 1,10-phenanthroline monohydrate were purchased from Sigma-Aldrich (St. Louis, MO, USA), and ammonium hydroxide (25%), sodium hydroxide, hydrochloric acid (37%), hydroxylamine hydrochloride, ammonium acetate and acetic acid were obtained from Vekton (St. Petersburg, Russia). All reagents had a “chemically pure” degree of purity.

### 3.2. Synthesis and Characterisation of Magnetic Nanoparticles

Magnetite particles were prepared using a modified method of co-precipitation of iron (II, III) chlorides (at a molar ratio of 1:2) in an alkaline ammonia medium [37]. All stages of the synthesis were carried out in an inert argon atmosphere to prevent the oxidation of Fe^2+^ and oleic acid. FeCl_2_·4H_2_O (1.3 g) and FeCl_3_·6H_2_O (3.5 g) were dissolved in 30 mL of deionised water and preheated to 90 °C. Then, 9 mL of 25% NH_4_OH was quickly added to this solution with vigorous stirring, and the resulting suspension of magnetite particles was stirred for another 15 min. Magnetite particles were purified using magnetic decantation and washed 10 times with deionised water:ethanol (at a volume ratio of 4:1). After every two washings, the magnetic particles were subjected to ultrasonic treatment (35 kHz, 200 W) for 20 min.

To prepare a stabiliser, 0.16 g of NaOH was dissolved in a solution containing 2 mL of deionised water and 3 mL of ethanol. Then, 1.75 mL of oleic acid was added to the alkaline solution, and the mixture was stirred. Thus, a solution containing sodium oleate and free oleic acid was obtained.

The freshly prepared aqueous alcoholic stabiliser was added to the purified magnetite. The resulting suspension was stirred intensively at 100 °C for 1 h. During the thermal treatment, ethyl alcohol was evaporated. Lastly, the suspension was centrifuged for 10 min at 4830× *g* to remove large aggregates.

The sizes and morphology of the obtained particles were investigated using transmission electron microscopy (TEM) on a FEI Tecnai Osiris microscope (USA) and dynamic light scattering (DLS) on a NANO-flex analyser (Microtrac Inc., Krefeld, Germany). The ζ-potential of the particles was measured on a Stabino analyser (Microtrac Inc.).

The quantitative determination of iron in the obtained magnetic suspension was carried out using a photocolourimetric method. The method is based on the interaction of Fe^2+^ ions with 1,10-phenanthroline with the formation of an orange-coloured complex [55].

The magnetisation of the samples was measured by the induction method, using an EZ11 vibration magnetometer (Microsense Inc., Lowell, MA, USA) at room temperature (~25 °C).

Studies on the heating of magnetite nanoparticle suspensions (Fe_3_O_4_-OA-NP) under an alternating magnetic field (AMF) were carried out using a special device produced by Pharmag (AMT&C Group, Troitsk, Russia) [56]. This device generates an AMF with a frequency of 100 kHz and a maximal strength amplitude of 8 kA/m. The working area was the inner cavity of the solenoid with a diameter of 70 mm and a height of 80 mm. A magnetic suspension (0.5 mL at a final concentration of 112 mg Fe/mL) was placed inside a copper coil and exposed to an AMF (8 kA/m, 100 kHz). The *SAR* value was calculated using the following formula:

$$SAR=C\frac{M}{m}\frac{dT}{dt},$$

where *C* is the specific heat of water (4190 J kg^–1^ K^–1^), *dT*/*dt* is the time derivative of the temperature calculated at the initial part of the heating curve, *M* is the mass of the suspension, and *m* is the mass of the nanoparticles [57].

The stability of the resulting Fe_3_O_4_-OA-NP suspension was studied for 18 months. The stability was evaluated using the change in the hydrodynamic diameter and ζ-potential of the nanoparticles. The SAR value of the magnetic fluid was also monitored during this time.

### 3.3. Animals

White inbred male BALB/c mice aged 45–50 days and weighing 18–22 g (*n* = 66) and white Wistar rats aged 8–9 weeks and weighing 225–325 g (*n* = 35) were used for the experiments. All animals were purchased from the «Stolbovaya» nursery «Scientific Center for Biomedical Technologies of the Federal Medical and Biological Agency» (Russia). The animals were fed standard laboratory chow and water ad libitum. The containment environment had a constant humidity of 40–60% and a temperature of 20–22 °C with a 12/12 h light/dark cycle.

Experiments with the animals were carried out in accordance with the rules for working with animals formulated by Directive 2010/63/EU of the European Parliament and of the Council of the European Union on the protection of animals used for scientific purposes and were approved by the local ethics committee at the Medical Institute of National Research Mordovia State University, approval date: 9 May 2020, approval code: 88.

### 3.4. Acute (Single-Dose) Toxicity of Fe_3_O_4_-OA-NP

Acute toxicity was assessed as the toxicity of the test substance (Fe_3_O_4_-OA-NP) after a single dose [58]. It was studied in mice using two routes of administration: intramuscular and intraperitoneal.

#### 3.4.1. Intramuscular Administration

When administered intramuscularly, the substance was injected once into the anterior and posterior muscle groups of both thighs (0.5 mL per limb) [58,59]. The study was carried out using the maximum possible volume with an intramuscular injection, which is 1 mL per mouse. The study included six mice. Since this dose did not induce toxic effects and deaths, lower doses were not studied.

#### 3.4.2. Intraperitoneal Administration

To study the toxicity of an intraperitoneal injection of Fe_3_O_4_-OA-NP, 10 groups of mice (6 mice each) were formed. The investigated substance was injected intraperitoneally once at increasing doses of 150, 300, 450, 600, 750, 900, 1200, 1500, 1800 and 2100 mg Fe/kg. The survival and behaviour of the animals were recorded daily. The dead animals were dissected. The surviving animals were observed for 30 days and then euthanised. The lungs, heart, liver, kidneys, part of the small intestine and abdominal wall were collected from fallen and surviving mice for histological examination (described in the Supplementary Materials, Section S1). Toxicity parameters were determined via probit analysis using Probit^®^ NCSS Statistical Software 2021 (CA, USA). Haematological and biochemical blood parameters were also investigated (described in the Supplementary Materials, Section S2).

### 3.5. Study of Local Irritant Action

The local irritant effect upon the administration of Fe_3_O_4_-OA-NP was studied in rats, since the anti-tumour effect of HT was supposed to be studied in a model of solid cancer in rats. This study was performed on seven rats. Fe_3_O_4_-OA-NP was injected intramuscularly into the right hind paw of the hip flexor muscle group. The volume of the injected fluid was 2 mL/kg. The total volume of the suspension was divided into five equal parts, which were injected at equal distances from one another. The total dose of injected magnetite was 233 mg Fe/kg. This dose is considered appropriate for MHT [32]. The rats were euthanised on the seventh day of the experiment. Histological examination of the muscles from the injection site and the contralateral (intact) limb was performed.

### 3.6. Antineoplastic Effect and Safety of MHT

#### 3.6.1. Establishment of Animal Tumour Model

The study was performed with Wistar rats. A tumour strain of W256 carcinosarcoma was used for this study (N.N. Blokhin Russian Cancer Research Centre). Under sterile conditions, 8-day-old W256 carcinosarcoma tumour cells were crushed to a suspension state and washed with Medium-199 to remove blood. Then, the tumour cell suspension was implanted in the right thigh area subcutaneously at a volume of 0.2 mL (1 × 10^6^ cells/mL). The Walker 256 tumour is characterized by aggressive growth and metastasis, comparable to the activity of human cancer, and is often used in experiments using physical and chemotherapeutic factors. In particular, it is widely used to assess the effects of local hyperthermia [60].

#### 3.6.2. Experimental Groups

On the fourth day after the implantation of tumour cells, the rats were randomly divided into four groups of seven animals each.

In the first group, animals with tumours did not receive any treatment (control group). In the second group, starting from the fourth day, the animals were exposed to a magnetic field for 30 min twice, with an interval of 24 h (magnetic field group). In the third group, 0.5 mL of Fe_3_O_4_-OA-NP was injected once into the tumour (ferrofluid control group). In the fourth group, after intra-tumoural injection of Fe_3_O_4_-OA-NP, the animals were exposed to a magnetic field for 30 min twice, with an interval of 24 h (MHT group).

#### 3.6.3. Induction of HT

All Fe_3_O_4_-OA-NP-induced MHT experiments in vivo were carried out safely using the aforementioned (100 kHz, 8 kA/m) original heating device (Pharmag, Russia). The rats were placed in the induction coil using a specially designed fluoroplastic supporter, such that the tumours were located exactly in the region with the highest field density. The heating temperature of the tumour surface and the surrounding skin was monitored using a Seek Thermal Compact PRO thermal imaging camera (Santa-Barbara, CA, USA). The strength amplitude of the magnetic field was varied in the range of 5–8 kA/m to maintain a skin temperature above that of the tumour node in the diapazone (44–46 °C), which is equal to the temperature inside the node (approximately 46–48 °C). Data on this temperature difference were obtained from a preliminary experiment, in which we compared two methods of tumour node temperature control. Under conditions similar to those used in our experiment on tumour-bearing animals, we controlled the temperature inside the tumour node using a thermocouple and the external temperature of the skin above the node using an infrared imaging camera. The temperature difference inside and outside the node was 2.6 ± 0.3 °C, with a correlation coefficient of R = 0.979. The measurement results are given in the supplementary material (Figure S4).

#### 3.6.4. Assessment of Tumour Growth and Rat Survival Time

Animal tumour volumes were measured initially on the day of treatment and then every two days thereafter. A digital calliper was used to measure the longest (*a*) and shortest diameter (*b*) of a given tumour. Tumour volume was calculated using the formula V = (πab^2^) × (π/6) [61]. Subsequently, tumour growth curves were plotted for each group of rats. In addition, the average lifespan in each group was estimated.

The Perls’ staining method was used to identify iron oxide nanoparticles in the tumour tissue. This method allows the detection of Fe^2+^. With this method, in the case of intracellular localization of iron-containing particles, light microscopy makes it possible to identify dark blue Prussian blue granules on the background of cell contours, while the intercellular space remains unstained [62].

### 3.7. Euthanasia

The animals were euthanised under general anaesthesia using Zoletil (Virbac, France) and Rometar (Bioveta, a. s., Ivanovice na Hané, Czech Republic). Mice were euthanised by cervical dislocation. Rats were euthanised by decapitation.

### 3.8. Statistical Analysis

SPSS 10.0 software was used to analyse the data obtained. The data are expressed as the mean ± standard deviation. ANOVA was used for multiple comparisons; data with abnormal distribution were analysed using the nonparametric Mann–Whitney U-test. The Kaplan–Meier log-rank test was used to calculate survival rates. The significance level was set at *p* < 0.05.

## 4. Conclusions

In this study, we prepared a colloidal suspension of iron oxide nanoparticles stabilised with oleic acid/sodium oleate for MHT to cure tumours. The properties of the synthesised magnetic fluid, which make it exert therapeutic effects, are the high concentration of iron oxide and an SAR value acceptable for MHT. Histological and biochemical analysis of the iron oxide nanoparticles showed almost no toxicity when administered intramuscularly at the maximum dose limited by the volume of the injected colloidal suspension. For this administration route, there were no pathomorphological changes in the internal organs, and the local irritant effect was minimal. These data can be extrapolated to all variants of regional extravascular administration, including intra-tumoural injection. This pathway was used to study the effectiveness of MHT with the synthesised fluid in rats with transplanted W256 carcinoma.

The effectiveness of MHT assessed by the survival rate of animals with tumours in this experiment was 85%. It should be noted that the physicochemical properties of the developed fluid at an SAR value of 13 W/g and concentration of 112 mg Fe/mL enable sufficient heat release at a low frequency of 100 kHz and field of 8 kA/m, which makes it possible to fabricate a relatively small-sized device for MHT for humans. In theory, if we take into account the histological picture, which shows phagocytes that actively absorb magnetic fluid and good distribution of fluid in fatty structures, then we can assume that this type of colloidal magnetite is biodegradable. If so, then the liquid does not require operations to remove nanoparticles after HT for a hard-to-reach localisation, which is essential for brain tissue after treatments for glioblastoma. Therefore, the proposed fluid is a potential therapeutic agent for tumour HT.

## Figures and Tables

**Table 1:** Toxicity parameters for Fe_3_O_4_-OA-NP magnetic fluid when administered intraperitoneally.

Parameter	Dose, mg Fe/kg	95% Confidence Intervals Limits	
		Lower	Upper
LD_16_	183.8	20.6	347.4
LD_50_	652.3	343.2	1044.8
LD_84_	2315.5	1340.3	8488.8

## Data Availability

Data confirming the results of the study can be obtained upon request from the authors at the Medical Institute of Ogarev Mordovian State University. E-mail: oleg-kulikov-84@mail.ru.

## Supplementary Materials

The following supporting information can be downloaded at: https://www.mdpi.com/article/10.3390/ijms23084234/s1.
