# Supplementary material for: Magnetic Hyperthermia Nanoarchitectonics via Iron Oxide Nanoparticles Stabilised by Oleic Acid: Anti-Tumour Efficiency and Safety Evaluation in Animals with Transplanted Carcinoma
Source: Int J Mol Sci. 2022 Apr 11;23(8):4234. doi: 10.3390/ijms23084234 (PMC9025391; doi:10.3390/ijms23084234)
Supplement: Supplementary file 1 [file ijms-23-04234-s001.zip › ijms-1673838-supplementary.pdf]

Supplementary material

Table. S1. Manifestation and mortality of mice after intraperitoneal administration Fe_3O_4-OA-NP and toxicity parameters

Groups	Dose, mgFe/kg	Dose, ml/kg	Appetite	Activity	Mortality
1	150	1,4	Good	Active	1/6
2	300	2,7	Good	Active	0/6
3	450	4,1	Good	Active	2/6
4	600	5,5	Good	Active	2/6
5	750	6,8	Good	Active	3/6
6	900	8,2	Good	Active	5/6
7	1200	10,9	Worse	Passive	4/6
8	1500	13,6	Worse	Active	4/6
9	1800	16,4	Worse	Passive	5/6
10	2100	19,1	Worse	Passive	6/6

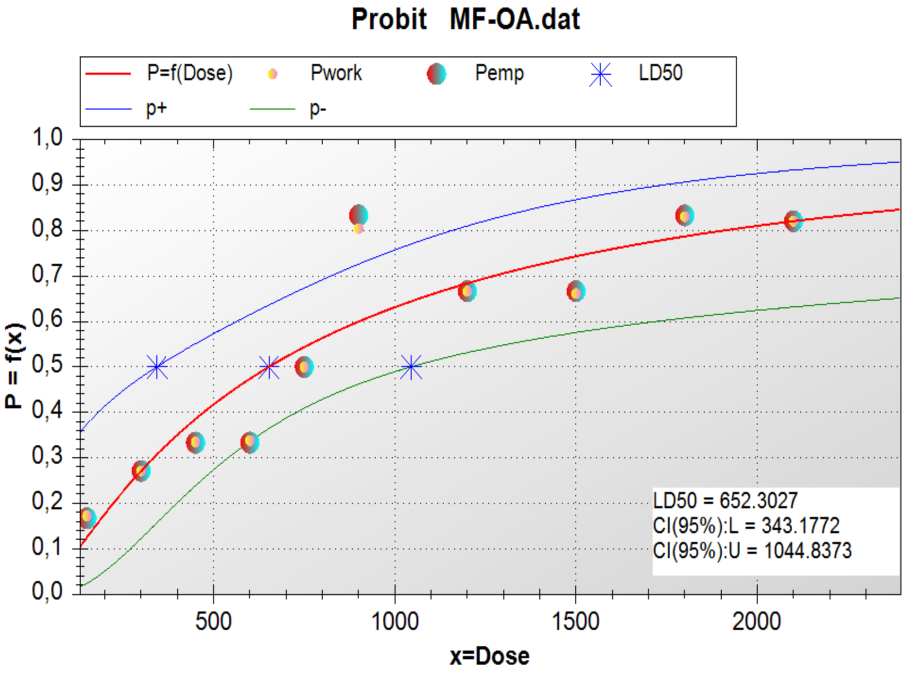

Figure S1. Dose-lethality ratio for magnetic fluid Fe_3O_4-OA-NP when administered intraperitoneally.

1. Histopathological analysis in mice

1.1. Methods of histopathological analysis

Histological examination was performed for surviving animals in the group that received 0.3 ml (900 mgFe/kg, ~1.5 LD_{50}) of the Fe_3O_4-OA-NP magnetic fluid intraperitoneally and for animals that received 5600 mgFe/kg of Fe_3O_4-OA-NP intramusculary.

Organ, muscle, and tumour tissues were fixed in 10% buffered formalin. The preparations were embedded in paraffin, and sections (7–8-µm thick) were cut using a PFM Rotary 3003 microtome (Germany). The dewaxed sections were exposed to the Perls' histochemical reaction (the formation of Prussian blue) to detect iron in the

tissues. Background staining of the sections was performed with haematoxylin and eosin (Khimprom, Russia). Histological preparations were examined under a Nikon Eclipse NI-SS light microscope (Japan). Micrographs were taken using a Nikon DS-F21 attachment (camera) (Japan) at magnifications of 100×, 200×, and 400×.

1.2 Result of histopathological analysis after intraperitoneal injection of Fe_3O_4-OA-NP in mice

On macroscopic assessment, the abdominal organs had a brown staining of the outer surface, but no visible macroscopic changes were noted in the section. The organs of the chest cavity (heart and lungs) did not show visible pathology. The results of microscopic assessment are shown in Figure S2.

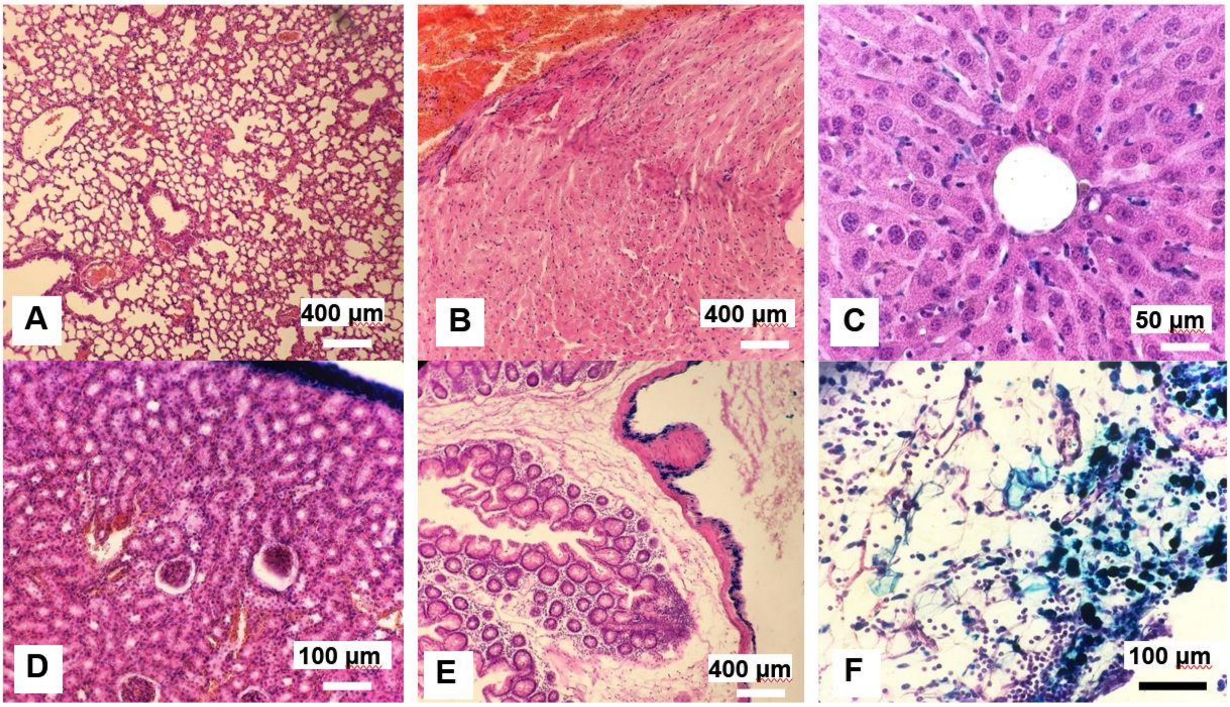

Figure S2. Histological changes in the lung (A), heart (B), liver (C), kidney (D), small intestine (E), and large intestinal omentum (F) of mice on the 30th day after intraperitoneal injection of Fe_3O_4-OA-NP magnetic fluid with a magnetite dosage of 900 mgFe/kg. The magnetic fluid is blue in colour and is visualised inside cells and as free clusters. There are no disorders in the lungs, and magnetic fluid is not detected (A). The heart muscle of the mice showed no pathological changes, and the Perls' reaction was negative (B). In the sinusoidal capillaries of the liver, magnetite is visualised, and is diffusely located, with a positive Perls' reaction (C). The Perls' reaction indicates the presence of magnetite in the peritoneum, which covers the kidney and intestines (D, E). The Perls' reaction shows the accumulation of magnetite nanoparticles in the cytoplasm of the omentum phagocytes (F).

The alveolar structure in the lungs (Fig. S2 A) was completely preserved. The vessels were not spasmodic. All bronchi were dilated. We did not find toxic phenomena such as oedema of the interalveolar septa or vascular thrombosis. The Perls' reaction in the micropreparations of the lungs was negative, which indicated the absence of penetration of Fe_3O_4-OA-NP into the pulmonary blood flow.

The liver tissue of mice had a lobular structure (Fig. S2 C); signs of dystrophy and necrosis of hepatocytes were absent. The blood supply was reduced. Blue aggregations of Fe_3O_4-OA-NP were visualised around the central veins of the liver

lobules. On detailed examination with a magnification of 400×, parietal Kupffer cells with iron compounds in the cytoplasm were discovered in the sinusoidal capillaries of the liver. They were visualised as blue granules that were tightly adjacent to each other.

The renal tissue of the mice had a pronounced plethora of the cortex. Glomerular and tubular structures were completely preserved. The epithelium of the tubules did not show signs of dystrophy. The capsule of the glomeruli was of the usual form, without signs of expansion. The visceral peritoneum covering the kidneys was blue, as it was impregnated with iron oxide nanoparticles. The magnetic fluid did not penetrate the renal tissue (Fig. S2 D).

The intestines of the mice showed no pathological changes but contained food chyme and had accumulated leucocytes in the lumen (Fig. S2 E). The muscular wall had zones of thickening and thinning. This indicated the absence of violations of the functional state of the intestine, the presence of peristalsis, and digesting activity. The peritoneal layer covering the intestine was coloured blue because of the accumulation of iron oxide nanoparticles.

The large omentum contained large numbers of macrophages and granulocytes. The cytoplasm of the omentum leucocytes was blue because of the content of iron oxide nanoparticles. It is possible that intraperitoneal injection of Fe_3O_4-OA-NP caused abundant migration of phagocytes into the omentum tissue. Adipocytes of adipose tissue had an intense blue coloration of the intercellular septa, probably due to the high lipophilicity of the injected magnetic fluid (Fig. S2 F).

1.3. Result of histopathological analysis after intramuscular injection of Fe_3O_4-OA-NP in mice

After the intramuscular injection of Fe_3O_4-OA-NP, there were no signs of disturbance in the morphology of the internal organs of mice during macro- and microscopic assessment (Fig. S3).

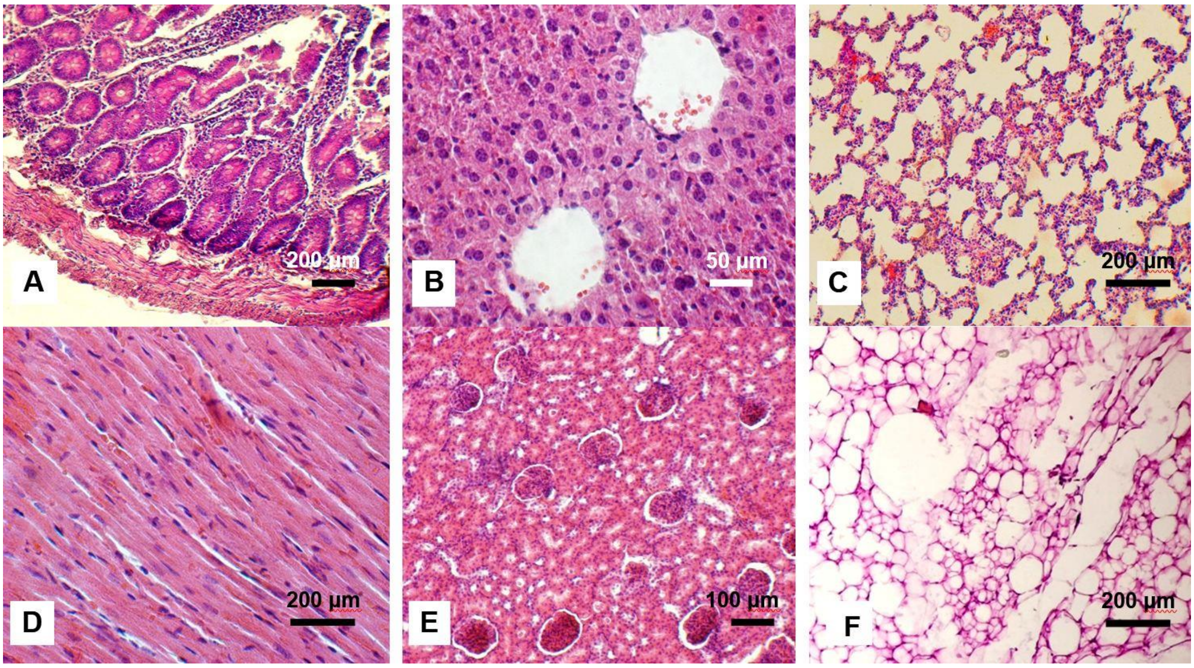

Figure S3. Histology of internal organs of the mice: small intestine (A), liver (B), lung (C), heart (D), kidney (E) and large intestinal omentum (F) of mice on the 30th day after intramuscular injection of magnetic fluid Fe_3O_4-OA-NP in a single dose of 5600 mgFe/kg.

2. Haematological and biochemical blood tests in rats

2.1. Method of haematological and biochemical blood tests

Blood samples were collected from the tail vein. The collected blood was added to heparin- and EDTA-coated blood collection tubes for blood cell count and blood chemistry analysis, respectively. Blood cell counts were measured within 1 h after collection. Haematological parameters included haemoglobin (HGB), red blood cell (RBC), and white blood cell (WBC) counts, as well as neutrophil (NEU), lymphocyte (LYM), and monocyte (MON) counts. All haematological parameters of the blood samples were determined using a haematology analyser, PCE-90Vet (HTI Inc., USA).

For blood chemistry analyses, blood samples were briefly centrifuged at 3000 rpm for 15 min to obtain plasma. The following parameters were determined within 24 h post collection using a blood chemistry analyser, XL-200 (Erba Lachema, Czech Republic): bilirubin (BIL), alkaline phosphatase (ALP), alanine aminotransferase (ALT), aspartate aminotransferase (AST), blood urea (BU), glucose (GLU), creatinine (CRE), serum iron (SFe), and lactate dehydrogenase (LDH).

2.2. Results of haematological and biochemical blood tests

The absolute values of these indicators are shown in Table S2 – S5.

Table S2 Indicators of a general blood test in rats against the background of intramuscular injection of Fe_3O_4-OA-NP at a dose of 233 mgFe/kg

Indicators	Significance at the stages of observation		Changes, %	Validity of differences
	Before injection	7 days after injection		
HGB (g/l)	135±3	125±12	- 7,4	0,097668
RBC ($\times10^{12}$)	6,81±0,36	6,75±0,47	- 0,9	0,819179
WBC ($\times10^{9}$)	9,87±3,77	7,59±3,97	-23,2	0,007255
NEU ($\times10^{9}$)	1,63±0,86	0,91±0,71	-43,9	0,004015
LYM ($\times10^{9}$)	7,01±2,65	5,91±2,97	-15,7	0,037873
MON ($\times10^{9}$)	1,23±0,47	0,76±0,40	-38,4	0,015517

Table S3 Indicators of biochemical blood test in rats against the background of intramuscular injection of Fe_3O_4-OA-NP at a dose of 233 mgFe/kg

Organ function or process		Significance at the stages of observation		Changes, %	Validity of differences
		Before injection	7 days after injection		
Liver	BR, µmol/l	2,43±0,61	2,63±0,60	8,1	0,6401
	ALT, U/l	170±22	168±10	-1,3	0,7280
	AST, U/l	160±13	129±17	-18,9	0,0133
	ALP, U/l	1057±453	408±677	-61,4	0,0139
Kidney	BU, mmol/l	5,73±0,93	6,31±1,27	10,2	0,3846
	CRE, µmol/l	0,057±0,03	0,034±0,002	-41,3	0,07925
Iron	SFe, µmol/l	46,1±6,4	33,6±2,9	-27,2	0,0021

exchange					
Heart, Liver	LDH, U/l	755±289	794±418	5,1	0,8838
Carbohydrate metabolism	GLU, mmol/l	6,34±0,43	10,99±1,89	73,2	0,00038

There was no significant effect of the studied substance on the levels of haemoglobin and erythrocytes (although there was a tendency towards a decrease in haemoglobin levels). There was a significant decrease in the number of all types of leucocytes (neutrophil granulocytes, lymphocytes, and monocytes).

There were no changes in the levels of markers of liver, kidney, and heart damage. A decrease in the levels of AST and ALP can be regarded as a natural fluctuation of the indicator within normal values. There was a significant decrease in serum iron levels (27%) and an increase in glucose concentration (73%).

Thus, the study showed that the investigated substance is not hepato- or nephrotoxic, and most likely does not affect the heart. The revealed manifestations of haematotoxicity in the form of a decrease in leucocyte counts and serum iron levels can be artifacts (a small number of observations, one dose) or real side effects. The same can be said for the glucose levels. Additional studies are needed to clarify the mechanisms underlying these changes.

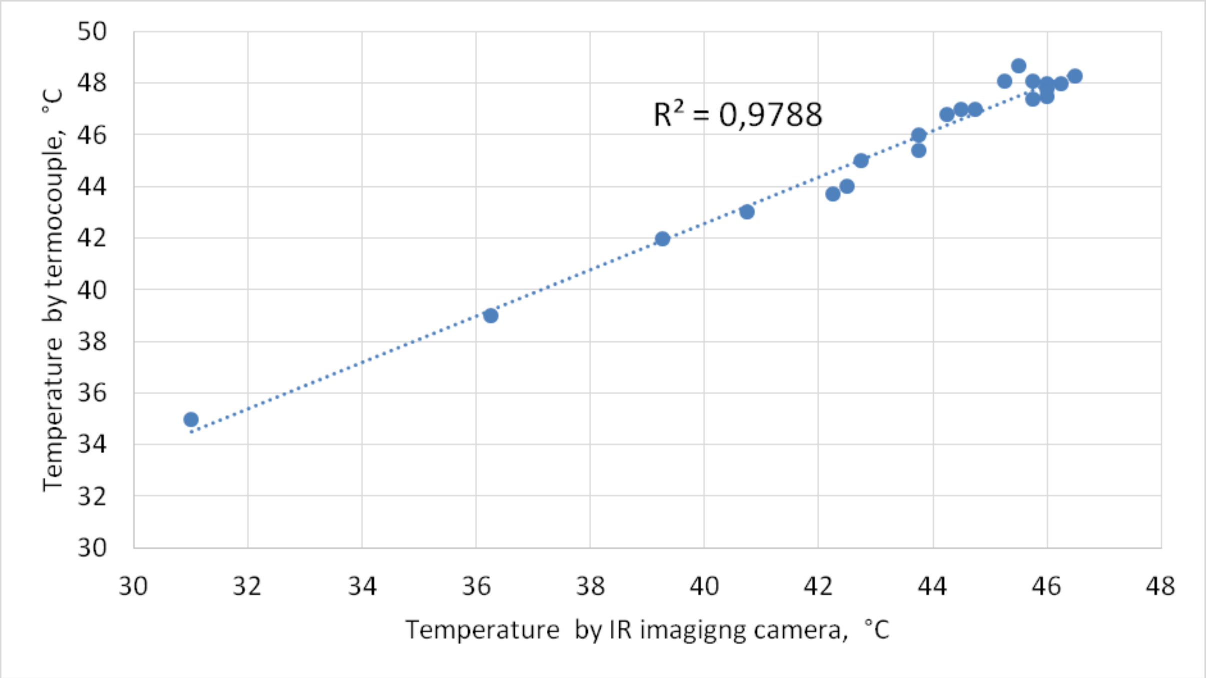

Figure S4. Correlation of the growth of tumor temperature measured with the insertion of a thermocouple and with an infrared camera. The detectors data correlated with a coefficient of +0.98.

Table S4 Indicators of a complete blood count against the background of tumor growth and hyperthermia, 19th and 40th day from the moment of tumor transplantation

Indicator	day	Experimental groups				
		Control W256	W256+MF	W256+MNPs	W256+MF+MNPs	no tumor
HGB, g/l	19	69.6±21.6 [a]	58.8±5.7 [a]	71.3±12.4 [a]	107.0±12.9 [ac]	134.0±3.0
	40	-	-	-	115.7±6.4 [acd]	
RBC, $\times 10^{12}$/l	19	3.5±1.0 [a]	2.8±0.3 [a]	3.5±0.7 [a]	5.9±0.8 [c]	6.5±0.3
	40	-	-	-	6.5±0.3 [bcd]	
WBC, $\times 10^{9}$/l	19	37.2±9.4 [a]	24.7±3.7 [a]	21.4±5.2 [a]	16.9±6.4	9.9±4.5
	40	-	-	-	4.2±1.1 [bcde]	
NEU, $\times 10^{9}$/l	19	12.0±6.0 [a]	9.3±3.2 [a]	5.2±0.4 [a]	1.8±0.2	1.7±0.8
	40	-	-	-	0.7±0.1 [cde]	
LYM, $\times 10^{9}$/l	19	18.7±4.5 [a]	6.0±2.3 [b]	12.0±2.8	9.8±3.5	7,0±2.7
	40	-	-	-	2.8±0.6 [abde]	
MON, $\times 10^{9}$/l	19	6.6±2.8 [a]	3.8±1.1 [a]	4.2±0.6 [a]	5.3±3.2 [a]	1.2±0.4
	40	-	-	-	0.7±0.2 [bcd]	

[a]P<0.05 vs group without tumor; [b]P<0.05 vs group control W256; [c]P<0.05 vs group W256+MF; [d]P<0.05 vs group W256+MNPs; [e]P<0.05 vs group W256+MF+MNPs (19day); MF- magnetic field; MNPs - magnetic ferrofluid.

Table S5 Indicators of biochemical blood test against the background of tumor growth and hyperthermia, 19th and 40th day from the moment of tumor transplantation

Indicator	day	Experimental groups				
		Control W256	W256+MF	W256+MNPs	W256+MF+MNPs	no tumor
ALT, U/l	19	61.4±21.3 [a]	35.8±6.5 [a]	60.7±3.3 [a c]	112.5±13.0 [cd]	161±28
	40	-	-	-	105.5±9.8 [cd]	
AST, U/l	19	382.4±65.8 [a]	302.2±60.2 [a]	189.3±4.5	259.8±87.7	163±23
	40	-	-	-	234.7±30.0	
BR, μmol/l	19	5.1±0.1	6.7±0.9	5.9±1.5	8.8±1.8 [ab]	5.2±0.1
	40	-	-	-	9.3±1.1 [ab]	
BU, mmol/l	19	22.0±5.8	8.5±0.8 [b]	5.5±0.6	26.4±17.6 [a]	5.7±0.8
	40	-	-	-	8.2±1.0 [ab]	
CRE, μmol/l	19	0.06±0.00	0.06±0.00	0.05±0.00	0.10±0.04 [a]	0.04±0.00
	40	-	-	-	0.05±0.0	

[a]P<0.05 vs group without tumor; [b]P<0.05 vs group control W256; [c]P<0.05 vs group W256+MF; [d]P<0.05 vs group W256+MNPs; MF- magnetic field; MNPs - magnetic ferrofluid.
